# Larynx Injuries After Orotracheal Intubation: What are Risk Factors Observed in Patients with Covid?

**DOI:** 10.1055/s-0045-1812320

**Published:** 2026-03-03

**Authors:** Leticia Felix, Gabriel de Souza Mares, Lara Freire Bezerril Soares, Romualdo Suzano Louzeiro Tiago, Sergio Roberto Nacif

**Affiliations:** 1 Department of Otolaryngology, Hospital do Servidor Público Estadual de São Paulo (HSPE), São Paulo, SP, Brazil; 2 Department of Pulmonology, Hospital do Servidor Público Estadual de São Paulo, São Paulo, SP, Brazil

**Keywords:** COVID-19, SARS-CoV-2, laryngostenosis, larynx

## Abstract

**Objective:**

To evaluate the risk factors for the development of laryngotracheal injuries in patients with coronavirus disease 2019 (COVID-19) undergoing orotracheal intubation (OTI).

**Methods:**

A cohort study was performed with intubated patients diagnosed with COVID-19, hospitalized from March 1st to October 31st, 2020. They were called for outpatient follow-up after being discharged from the hospital.

**Results:**

There were 421 patients with COVID-19 (31%) who required OTI, of which the outcome was: hospital discharge for 172 (40.9%) and death for 249 (59.1%). Outpatient videoendoscopy was performed in 95 patients (55.2%).

**Conclusion:**

We observed a greater risk for the development of laryngotracheal injury in patients who presented an increase in leukocyte count at hospital admission with lymphopenia, hypoalbuminemia, increased arterial lactate, troponin, and total bilirubin, as well as endotracheal tube with larger caliber and pronation. Furthermore, patients who at the time of OTI showed greater inflammatory reactivity or developed coagulation disorders were also at greater risk.

## Introduction

Upper airway complications after orotracheal intubation (OTI) were considered a result of tissue ischemia due to endotracheal cuff pressures that compromise mucosal blood flow. 1 Although generally rare, they still constitute an important factor in morbidity after OTI, including damage to vocal cords, laryngotracheal stenosis or granuloma formation, and impaired swallowing. 2 3 4

Predisposing factors already described in the literature for the development of such complications include some intrinsic to the patient, such as comorbidities and immunity, as well as extrinsic factors, such as OTI tube size, excessive cuff pressure, and prolonged intubation before tracheostomy. 5 6 7 8

In the context of the coronavirus disease 2019 (COVID-19) pandemic, the severe acute respiratory syndrome coronavirus 2 (SARS-CoV-2) virus's rapid spread has led to an unprecedented increase in the number of patients requiring prolonged intensive care unit stays. 9 About 5% of individuals with this disease progress to severe conditions requiring mechanical ventilation. 10 In adults requiring mechanical ventilation, due to moderate-to-severe hypoxemic respiratory syndrome, the pronation position for 12 to 16 hours has been used, and it is often used in severe cases of COVID-19. 11 The goal of the present study was to evaluate the risk factors for the development of laryngotracheal injuries in COVID-19 patients, with severe clinical status, undergoing OTI.

## Methods

The present study was evaluated and approved by the Research Ethics Committee of the institution (protocol No. 37097920.9.0000.5463). According to the Declaration of Helsinki, written informed consent was obtained from each patient or a legal representative in case of mental incapacity.

A prospective cohort study was conducted with consecutive COVID-19 patients diagnosed by a reverse transcription polymerase chain reaction (RT-PCR) molecular test, hospitalized in a tertiary hospital from March 1 to October 31, 2020, who required OTI. The procedure was performed by the rapid response team of the institution, composed of the anesthesiology team. All patients submitted to OTI were evaluated regarding the outcome: discharge or death. Patients who had been discharged from the hospital were called for outpatient follow-up and endoscopic examination (video nasopharyngeal laryngoscopy and videolaryngoscopy). The videoendoscopy evaluation was performed between the 25 ^th^ and 185 ^th^ days, with an average of 100 and a median of 93 days.

Patients were excluded from this study if they had changes or laryngeal comorbidities prior to COVID-19 infection, were unable to perform the exam or come to the hospital, and/or did not have laboratory diagnosis by RT-PCR molecular test for COVID-19. For their evaluation, the following data were collected: presence of laryngotracheal injury; age (adults: < 60-years-old; and elderly: > 60-years-old); sex; OTI duration (days); comorbidities (diabetes mellitus, systemic arterial hypertension, and obesity); endotracheal tube (ETT) size; tracheostomy; indication of pronation during the OTI period; troponin, ferritin, potassium, D-dimer, leukocyte and lymphocyte count, platelets, hemoglobin, total bilirubin and lactic dehydrogenase (LDH), albumin, and arterial lactate from the date of hospital admission; use of corticosteroid therapy and use of anticoagulant during hospitalization; C-reactive protein, D-dimer, activated partial thromboplastin time (APTT), prothrombin time (TP), and international normalized ratio (INR) from the date of OTI.

Patients underwent endoscopic examination of the laryngotracheal segment with the use of a flexible rhinolaryngoscope, model 11101 RP2, (Karl Storz SE & Co. KG) and a laryngeal endoscope, model 8706 CA (Karl Storz SE & Co. KG). The monitor used was the Tele Pack X LED system (Karl Storz SE & Co. KG) which includes an endoscope, TP 45-1 06/2014/EW-PT. The exams were recorded in an Ultra USB 3.0 64 GB pen drive (SanDisk Corp., model SDCZ48-064G).

The steps of a laryngotracheal segment endoscopy were static evaluation of the larynx (supraglottis, glottis, and subglottis) and trachea during inspiration; maximum phonation time; and emission of vowels /e/ and /i/. The exams were analyzed by three laryngologists, without prior knowledge of patient data, independently. The diagnosis of the injury was obtained from the agreement of two or more examiners.

The data was tabulated in the Excel (Microsoft Corp.) program and submitted to statistical analysis with the Minitab Statistical Software (Minitab, LLC), version 19.1.1. Nominal qualitative variables were summarized through descriptive statistics. For analysis and outcome of categorical qualitative variables, the Pearson chi-square test was used. For analysis and outcome of the correlation of continuous variables, the binary logistic regression test was applied. The results were considered statistically significant when the *p-* value was lower than 0.05.

## Results

During the period of the present study, 1,357 patients were hospitalized with a diagnosis of COVID-19 confirmed by molecular RT-PCR test on nasal swab. The OTI for mechanical ventilation was required in 421 patients (31%). In patients submitted to this procedure, the outcomes found were hospital discharge in 172 (40.9%) and death in 249 (59.1%). Outpatient videoendoscopy evaluation was performed in 95 patients (55.2%). The group of patients who were discharged from hospital and were not examined represented 44.8% of the sample (n = 77). The reasons for absences were: 33 patients were unreachable (19.2%); 24 were not physically able to attend (14%); 15 refused the evaluation (8.7%); and 5 died after hospital discharge (2.9%). The average age of patients with COVID-19 submitted to OTI and evaluated by videoendoscopy was 59 years.

**Fig. 1:**
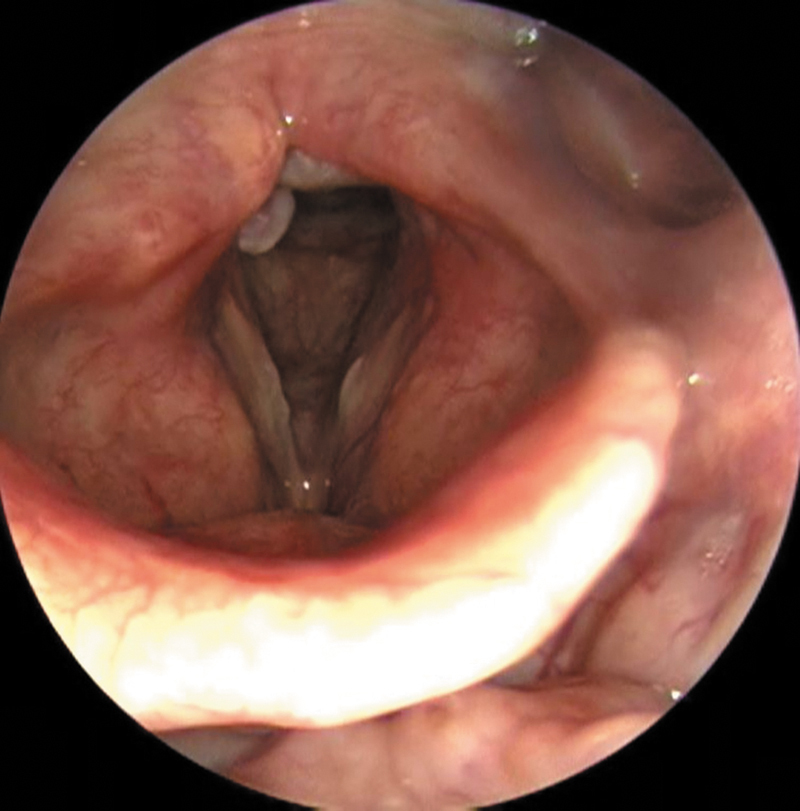
Laryngeal image on inspiration with granuloma in the right vocal process and hyperemia in the left vocal process.

**Fig. 2:**
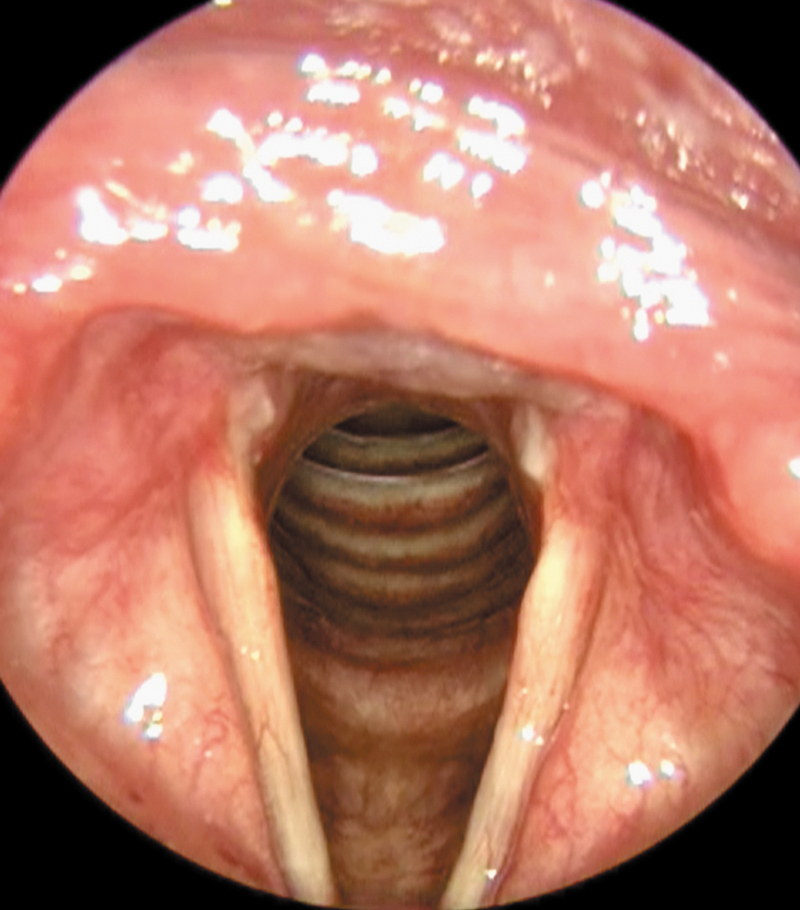
Laryngeal image on inspiration with stenosis in the posterior region of the glottis (type II) and granulation in bilateral vocal process.

The videoendoscopy evaluation was performed between the 25 ^th^ and 185 ^th^ day, with an average of 100 and a median of 93 days. For this evaluation, inflammatory injuries (hyperemia and granuloma) and scarring (stenosis and immobility) were considered. Laryngotracheal injuries were observed in 38 patients (40%), with the most diverse forms of presentation, such as hyperemia, granulomas, and stenosis. Laryngotracheal lesions were observed in 38 patients (40%), including hyperemia (6.3%), granulomas (15.8%), unilateral immobility (1%), and stenosis (16.9%), as shown in Figs. 1 , 2 .

Among the 421 patients who required ventilatory support in the intensive care unit, tracheostomy was performed in 27 between day 7 and 37 after OTI. In the group that received hospital discharge (n = 172), only 2 underwent tracheostomy (1.2%). These patients were evaluated, and no OTI-related injury was observed. Statistical significance was observed for the development of laryngotracheal injury to the following factors: increased leukocyte count; lymphopenia; total bilirubin, albumin, arterial lactate, and troponin from the date of hospital admission; endotracheal tube size; indication of pronation during intubation; and increased leukocyte count, D-dimer, prothrombin time (PT), and INR on the date of OTI ( Tables 1 2 3 4 ).

**Table 1:** Logistic regression data on the day of admission

Variables	OR	95% CI	*p* -value
Age	0.986	(0.958–1.016)	0.354
Platelets	10.019	(0.9972; 1.0067)	0.425
Total bilirubin	1.114870743	(357.5440–3.47632E + 07)	0.000
D-dimer	0.9459	(0.8462–1.0574)	0.251
Ferritin	10.002	(0.9997–1.0006)	0.433
Potassium	0.6237	(0.2890–1.3461)	0.208
Albumin	0.2217	(0.0782–0.6283)	0.001
LDH	10.042	(0.9996–1.0088)	0.065
Arterial lactate	17.021	(1.0156–2.8526)	0.038
AST	10.039	(0.9946–1.0132)	0.412
ALT	10.020	(0.9900–1.0142)	0.742
Troponin I	0.0000	(0.0000–0.0059)	0.004
Lymphocytes (%)	0.8430	(0.7627–0.9317)	0.000
PCR	10.396	(0.9958–1.0853)	0.072
Difference final–initial D-dimer	10.611	(0.9987–1.1274)	0.035
Hemoglobin	1.0476	(0.6861–1.5497)	0.829
Leukocytes	10.238	(0.9367–1.1191)	0.04

**Abbreviations:** ALT, alanine aminotransferase; AST, aspartate aminotransferase; CI, confidence interval; CRP, C-reactive protein; LDH, lactic dehydrogenase; OR, odds ratio; PCR, polymerase chain reaction.

**Table 2:** Logistic regression data on the day of intubation

Variables	OR	95% CI	*p* -value
D-dimer	1.030	(0.982–1.080)	0.219
INR	37.433789	(83.9308–1.669E + 07)	0.000
Leukocyte count	1.057	(1.002–1.154)	0.011
PCR	1.002	(0.979–1.025)	0.883
PT	2.4356	(1.360–4.352)	0.001
APTT	1.009	(0.962–1.059)	0.715
Fibrinogen	10.007	(0.9988–1.0026)	0.452
OTI days	0.958	(0.096–1.015)	0.131
ETT size			0.008
7.5 vs 7.0	0.521	(0.078–3.489)	
7.0 vs 8.0	2.768	(0.497–15.420)	
8.0 vs 7.5	5.314	(1.786–15.816)	

**Abbreviations:** APTT, activated partial thromboplastin time; CI, confidence interval; CRP, C-reactive protein; INR, international normalized index; ETT, endotracheal tube; OR, odds ratio; OTI, orotracheal intubation; PCR, polymerase chain reaction; PT, prothrombin time.

**Table 3:** Qualitative variables data on the day of hospital admission

Variables	Injury		*p* -value	
	Yes	No		
**Sex**			0.502	
Male	22 (23%)	29 (31%)		
Female	16 (17%)	28 (29%)		
**Age**			0.180	
Adults	20 (21%)	28 (29%)		
Elderly	18 (19%)	29 (31%)		
**Comorbidities**				
**SAH**			0.355	
Yes	23 (24%)	29 (31%)		
No	15 (16%)	28 (29%)		
**DM**			0.272	
Yes	12 (13%)	16 (17%)		
No	26 (27%)	41 (43%)		
**Obesity**			0.713	
Yes	19 (20%)	22 (23%)		
No	19 (20%)	35 (37%)		
**OTI** **days**			0.366	
Short	8 (8%)	9 (9%)		
Long	30 (32%)	48 (51%)		
**Heparinization**			0.844	
Intermediary	21 (22%)	30 (32%)		
Prophylactic	7 (7%)	10 (11%)		
Full	9 (10%)	17 (18%)		
**Corticosteroid** **therapy**			0.142	
Yes	27 (28%)	32 (34%)		
No	11 (12%)	25 (26%)		

**Abbreviations:** DM, diabetes mellitus; OTI, orotracheal intubation; SAH, systemic arterial hypertension.

**Table 4:** Qualitative variables on the day of intubation

	Injury		*p* -value
Variables	Yes	No	
Tracheostomy*			0.515
Yes	0 (0%)	2 (2%)	
No	38 (40%)	55 (58%)	
Leukogram*			0.027
> 11	16 (17%)	22 (23%)	
≤11	12 (13%)	45 (47%)	
CRP*			0.315
< 10	27 (28%)	30 (32%)	
≥10	16 (17%)	22 (23%)	
D-dimer*			0.014
Normal	2 (2%)	14 (15%)	
Changed	36 (38%)	43 (45%)	
APTT*			0.715
Normal	37 (39%)	56 (59%)	
Changed	1 (1%)	1 (1%)	
PT*			0.001
Normal	34 (36%)	56 (59%)	
Changed	4 (4%)	1 (1%)	
INR*			0.021
Normal	29 (31%)	53 (56%)	
Changed	9 (9%)	4 (4%)	
Pronation indication*			0.001
Yes	27 (28%)	20 (21%)	
No	11 (12%)	37 (39%)	

**Abbreviations:** APTT, activated partial thromboplastin time; CRP, C-reactive protein; INR, international normalized ratio; PT, prothrombin time.

**Note:** *Date of intubation.

## Discussion

Most laryngeal injuries after OTI have spontaneous resolution due to the regenerative capacity of the epithelium. However, under certain circumstances that lead to the aggravation of tissue perfusion and poor healing, the evolution of this process can get worse and lead to laryngeal injuries of variable gravity. 11 The main risk factors for postintubation laryngotracheal injuries described in the literature include: total duration, tracheal tube size, depth of sedation, and previous conditions (cardiovascular diseases, diabetes, smoking, and obesity). 12 13 14

In the present study, we evaluated patients who were admitted to an intensive care unit, with the need for OTI, all with a high degree of complexity and severe clinical status. Within this group, we searched for risk factors for postprocedure laryngotracheal injury, such as endotracheal tube size, pronation, and laboratory changes related to inflammation and coagulation.

The COVID-19 infection leads to deregulated immune response, generating leukocytosis at the expense of neutrophils and lymphopenia, explained by an immune suppression stage after the proinflammatory phase. 15 16 Studies have shown that the degree of lymphopenia and proinflammatory cytokine storm are independent risk factors for severe disease in this cohort. 17 18 In this study, we found a statistically significant correlation between increased leukocyte count and lymphopenia at the moment of hospital admission in patients who developed laryngotracheal injury after OTI, as well as increased leukocytes at the time of intubation ( Tables 1 , 2 , and 4 ). Ghosh et al. in a study with severe combined immunodeficient mice were able to demonstrate that circulating host systemic immune mediators, such as T and B leukocytes, play roles at the beginning of granulation tissue formation in response to tissue trauma in the airway. 19

Studies carried out on patients with COVID-19 suggest that the ventral position could be a risk factor for laryngotracheal complications. 4 20 21 22 In the present study, we observed that the pronation position during OTI was associated with the presence of laryngotracheal injuries ( Table 4 ). This position can cause ETT displacement, press the cuff on the tracheal walls, and generate ischemic injury to the laryngotracheal segment. 23 24 25 In the present study, according to institutional protocol, the cuff was inflated with a minimum volume to seal the trachea and with a maximum pressure of 30 cmH2O after OTI, with continuous monitoring.

The COVID-19 infection provides a prothrombotic and antifibrinolytic state, associated with increased concentrations of D-dimer and other fibrin degradation products, which were significantly associated with worse prognosis and higher mortality. 26 27 Coagulation alterations (increase in D-dimer throughout hospitalization, PT, and INR) were important prognostic factors for the development of laryngotracheal injury ( Tables 1 2 3 4 ). The prothrombotic state in COVID-19 determines microvascular injury, with potential development of ischemia and necrosis of the laryngotracheal mucosa. 20 28

Factors that can be modified (e.g., ETT size) are significantly associated with an increased risk of developing laryngotracheal injury after OTI. 29 30 In this study, based on the multivariable logistic regression model, we observed that the ETT number 7.0 was associated with a lower injury probability when compared to higher caliber tubes ( Table 2 ). In the pandemic scenario, it was suggested the use of a larger ETT (7.0–8.0 mm for women and 8.0–9.0 mm for men), with an inflated cuff to seal the airways before ventilation, which increases the risk of injury. 31 32 33

Patients with hypoalbuminemia during hospital admission due to COVID-19 had a higher risk and positive correlation for the development of laryngotracheal injuries ( Table 1 ), which has not been found in the literature so far. Albumin provides protection against inflammatory processes and the tissue damage associated with microcirculation. 34 35 The severity of hypoalbuminemia reflects the severity of inflammatory stress in acute and chronic states, since intact innate and adaptive immune responses depend on this protein. 36 37 Its interaction with bioactive lipid mediators can contribute to the occurrence of cytokine storms. 38

Studies that evaluated the severity of infection and mortality from COVID-19 observed that patients who evolved with more severe conditions presented the following changes: increased troponin, increased arterial lactate, and changes in liver function with increased total bilirubin. 16 39 40 41 In the present study, we observed that among OIT cases in severe clinical conditions, those who presented increased troponin, total bilirubin, and arterial lactate were associated with the development of laryngotracheal injury ( Table 1 ). Therefore, we present new risk factors not described in the literature for the development of laryngotracheal injuries in patients with COVID-19 undergoing OTI.

## Conclusion

We observed a higher risk for the development of laryngotracheal injury in patients who presented increased leukocyte counts with lymphopenia, increased total bilirubin, hypoalbuminemia, as well as increased arterial lactate and serum troponin at hospital admission. Patients who used higher-caliber endotracheal tubes and were submitted to pronation position, as well as those who presented higher inflammatory reactivity (increased leukocyte count) or developed coagulation disorders (increased D-dimer, PT and INR) at the time of OTI, were at higher risk for developing laryngotracheal injury.
